# The Hippo signaling pathway in leukemia: function, interaction, and carcinogenesis

**DOI:** 10.1186/s12935-021-02408-7

**Published:** 2021-12-25

**Authors:** Negar Noorbakhsh, Bentolhoda Hayatmoghadam, Marzieh Jamali, Maryam Golmohammadi, Maria Kavianpour

**Affiliations:** 1 Behbahan Faculty of Medical Sciences, Behbahan, Iran; 2 Department of Clinical Biochemistry, School of Pharmacy and Pharmaceutical Sciences, Isfahan University of Medical Sciences, Isfahan, Iran; 3 Gene Therapy Research Center, Digestive Diseases Research Institute, Tehran University of Medical Sciences, Tehran, Iran; 4 Applied Cell Sciences and Hematology Department, Faculty of Medical Sciences, Tarbiat Modares University, Tehran, Iran; 5 Department of Applied Cell Sciences, School of Advanced Technologies in Medicine, Tehran University of Medical Sciences, Tehran, Iran

**Keywords:** Hippo signaling pathway, Signaling, Leukemia, Hematologic neoplasms, Cancer

## Abstract

Cancer can be considered as a communication disease between and within cells; nevertheless, there is no effective therapy for the condition, and this disease is typically identified at its late stage. Chemotherapy, radiation, and molecular-targeted treatment are typically ineffective against cancer cells. A better grasp of the processes of carcinogenesis, aggressiveness, metastasis, treatment resistance, detection of the illness at an earlier stage, and obtaining a better therapeutic response will be made possible. Researchers have discovered that cancerous mutations mainly affect signaling pathways. The Hippo pathway, as one of the main signaling pathways of a cell, has a unique ability to cause cancer. In order to treat cancer, a complete understanding of the Hippo signaling system will be required. On the other hand, interaction with other pathways like Wnt, TGF-β, AMPK, Notch, JNK, mTOR, and Ras/MAP kinase pathways can contribute to carcinogenesis. Phosphorylation of oncogene YAP and TAZ could lead to leukemogenesis, which this process could be regulated via other signaling pathways. This review article aimed to shed light on how the Hippo pathway interacts with other cellular signaling networks and its functions in leukemia.

## Introduction

Hematological malignancies include lymphoma, myeloma, myeloproliferative neoplasms, myelodysplastic syndromes, and leukemia with several subtypes [1]. Leukemia is divided into lymphocytic and myeloid, which these two mentioned groups include acute and chronic groups. In total, leukemia is a clonal disorder that results from genetic and epigenetic changes in a hematopoietic stem or progenitor cells that disrupt main processes such as self-renewal, proliferation, and differentiation [2, 3]. Leukemic stem cells have several critical signaling pathways regulating stem or progenitor cell proliferation, hematopoiesis, self-renewal, tissue repair, and apoptosis [4, 5]. Cell numbers are based on signaling pathways that communicate extracellular and intracellular stimuli to gene transcription. For example, constitutive and cytokine-mediated activation of the PI3K/Akt/mTOR signaling pathway is a common hallmark in patients with acute myeloid leukemia (AML), and regulation of this system is a feasible therapeutic option in the treatment of AML [4, 6].

A new signaling pathway, Hippo, has played a crucial role in maintaining organ size by regulating cell proliferation and death in the last decade [7]. Due to the severe overgrowth phenotype, *Drosophila* mosaic genetic screens first found many mutations in the Hippo signaling pathway [8]. Because of its remarkable effectiveness in controlling organ size, as well as its apparent significance to tissue regeneration and cancer, the Hippo signaling pathway immediately has drawn widespread interest [9]. Mammalian sterile 20-like 1/2 (MST1/2, also known STK4/3), Salvador (SAV1), Large tumor suppressor homolog 1/2 (LATS1/2), MOB kinase activator 1A/B (MOB1a/b), and Yes-associated protein (YAP)/Transcriptional co-activator with PDZ binding motif (TAZ, also known WWTR1) are the mammalian orthologs of Hpo, Sav, Wts, Mats, and Yki, respectively [10].

Furthermore, a mutation in the genes encoding Hippo signaling proteins can cause significant organ shape or growth parameters [11, 12]. For example, renal cell carcinoma [13], pancreatic cancer [14], breast cancer [15], cholangiocarcinoma [16], medulloblastoma [17], and hepatocellular cell carcinoma (HCC) [18] have all been found to have an abnormal expression of YAP [19–21]. After analyzing 177 pairs of HCC, standard samples with comprehensive clinical data were matched; it was revealed HCC patients with YAP have an independent prognostic marker for overall survival and disease-free survival [22].

There is insufficient evidence to identify the tissue specificity and frequency of pathway components and YAP mutations in human leukemias [23]. Several hematological malignancies have been linked to abnormal expression or genetic deficiencies in the Hippo signaling pathway, including acute leukemia and lymphoproliferative neoplasms [24, 25]. For example, in a study by Chen et al. they measured the effects of YAP knockdown on HL-60 cells. Their study found that inhibition of YAP inhibits proliferation and induces apoptosis in the cell line [25]. YAP was also overexpressed in CML cells in Li et al.'s study and inhibiting this protein reduced CML cell growth, triggered apoptosis, and lowered the expression of YAP target genes c-Myc and survivin. As a result, YAP could play a key role in CML cell proliferation and leukemogenesis. The genetic or pharmacological suppression of YAP offers a potential CML therapeutic option [26].

The standard of care for leukemia depends on many factors chosen based on age and overall health, the type of leukemia, and the stage of the disease [27]. Common treatments used to fight leukemia include chemotherapy, targeted therapy, radiation therapy, bone marrow transplant, immunotherapy, and engineering immune cells. Despite advances and extensions in existing treatments, leukemia is associated with low survival rates and poor prognosis in some cases [28]. Some patients resist the usual treatments, and some relapse after remission induction [29]. Therefore, a search in this signaling pathway is needed to find a new treatment strategy. In this study, we intend to address the Hippo signaling pathway, its interaction with other pathways, and its importance in different types of leukemia; perhaps by providing important and effective proteins, this signaling pathway provides a novel treatment strategy for leukemia.

## Normal function of Hippo signaling pathway

**Fig. 1:**
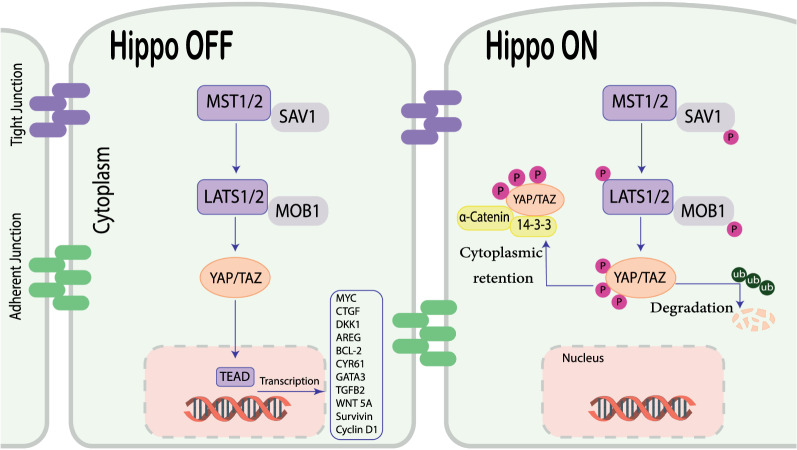
The core of the Hippo pathway. Multiple upstream signals regulate phosphorylation when the Hippo signaling pathway is activated, and MST1/2 kinases and SAV1 form a complex to phosphorylate and activate LATS1/2. YAP/TAZ proteins, two important downstream effectors of the Hippo pathway, are phosphorylated by LATS1/2 kinases. Phosphorylation of YAP/TAZ triggers the recruitment of 14-3-3 proteins, which promote cytoplasmic retention or proteolytic destruction. YAP/TAZ is not phosphorylated, localizes to the nucleus, forms a complex with transcription factor TEADs, and controls genes needed for endothelial cell proliferation, migration, and survival when the Hippo signaling pathway is turned off. *LATS1/2* large tumor suppressor kinase; *MST1/2* mammalian ste20-like kinase; *SAV1* scaffold protein salvador; *TAZ* transcriptional co-activator with PDZ-binding motif; *TEAD* TEA domain family member; *YAP* Yes-associated protein

The human Hippo pathway is based on a kinase signaling cascade including MST1 and MST2, as well as LATS1/2, SAV1 and MOB1, are two types of serine/threonine kinases [30]. When the Hippo pathway is inactive, unphosphorylated YAP/TAZ enters the nucleus and interacts with TEA DNA-binding proteins (TEAD1-4), then target genes regulated by this complex [31, 32] (Fig. 1). It is also proteolytically degraded when the Hippo pathway is activated [30, 33]. The Hippo pathway is dysregulated in cancer, enabling hyperproliferation, cellular invasion, metastasis, and chemoresistance [7, 34] (Fig. 1).

## Prognostic value of YAP in cancer

The level of YAP1 protein is elevated a variety of cancers, including colorectal cancer (CRC), gastric cancer, esophageal squamous cell cancer (ESCC), human hepatocellular carcinoma (HCC), osteosarcoma [35–38]. The Hippo pathway can be promoted by YAP1 influence across multiple signaling pathways. Many studies have recently examined how tumorigenesis, tumor growth,, epithelial to mesenchymal transition (EMT), resistance to apoptosis and cancer prognosis are all affected by YAP1 [39]. YAP1 facilitates the growth of tumor cells and can lead to a poor prognosis in many cancers. Additionally, YAP1, a tumor suppressor, has been identified as an apoptotic factor induced by DNA damage in collaboration with p73 and promyelocytic leukemia [40, 41]. In CRC, YAP expression was associated to TNM stage, and expression level of cyclin D1; Wang et al. found that YAP expression was also linked to a short overall survival (OS) [42].

Qu et al. reported that downregulating YAP inhibited cell migration and invasion, and YAP expression level could be a new marker for predicting the prognosis of patients with ESCC [36]. According to Xia et al. high levels of YAP expression were positively correlated with TEAD4 gene expression in ovarian cancer patients [43]. As Barry et al. reported, complete loss of YAP was associated with poorer patient survival and high-grade, stage IV disease than YAP-positive groups. Furthermore, they found that YAP could act independently to restrict Wnt signaling [44]. A meta-analysis assessed the relationship between YAP1 expression and overall survival (OS) in 20 studies that was conducted on 2067 patients. As a result of this study, it is statistically significant that positive YAP1 expression can negatively impact OS and disease-free survival (DFS) in patients with cancer. It’s also been claimed that YAP1 could behave as a tumor suppressor gene in some cancers, which would be a poor prognostic factor [45].

## The effect of Hippo signaling pathway in carcinogenesis

**Fig. 2:**
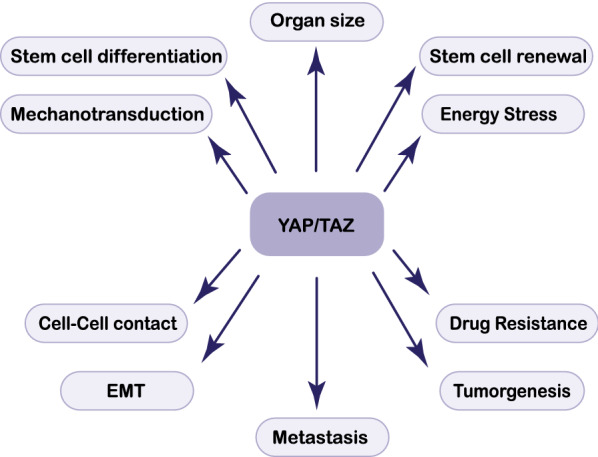
The Hippo pathway is dysregulated in cancer, enabling hyperproliferation, cellular invasion, metastasis, and chemoresistance. In these mechanisms, other signaling pathways affect the Hippo pathway that might lead to an increase or decrease YAP/TAZ complex level in the cytoplasm. *EMT* epithelial-mesenchymal transition; *Yap* Yes-associated protein

A wide range of upstream stimuli such as extracellular ligands, organ size, mechanotransduction, environmental stress, energy stress, and cell–cell contact controls YAP/TAZ activation in cancer cells [46]. The activation of YAP/TAZ via the dysregulation of the Hippo pathway is responsible for tumor development and confers cancer stem cell characteristics such as anoikis resistance, epithelial-to-mesenchymal transition, drug resistance, energy stress, and metastasis [47] (Fig. 2).

## Hippo signaling pathway and interaction with other signaling pathways

**Fig. 3:**
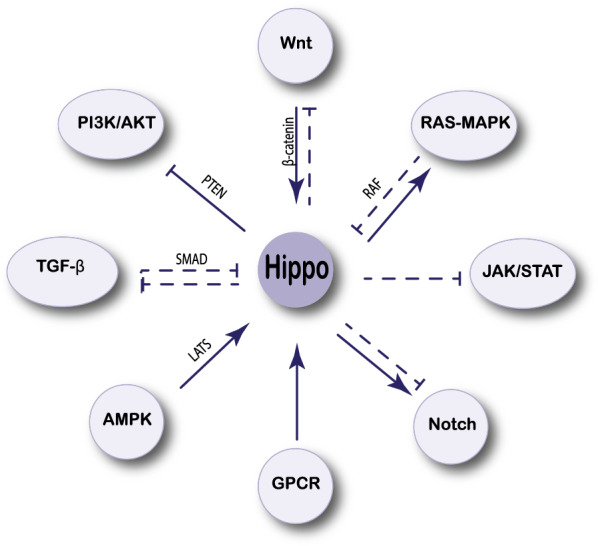
The Hippo pathway interacts with other signaling pathways. *Akt* protein kinase B; *AMPK* AMP-activated protein kinase; *GPCR* G protein-coupled receptor; *JAK-STAT* janus kinase and signal transducer and activator of transcription; *MAPK* mitogen-activated protein kinase; *PI3K* phosphatidylinositol-3-kinase; *RAS* rat sarcoma; *SMAD* mothers against decapentaplegic homolog; *TGF* transforming growth factor

Increased tissue development has been connected to YAP protein activation, as well as direct target genes like Myc, cell cycle regulators like CycE and E2F1, and apoptosis inhibitors like Diap1 and BIRC3, which have all been identified as contributory factors [48]. Other signaling pathways that may play a role in tissue growth control, including as the Wnt, Notch, EGFR, TGF, and Jak-STAT pathways, have also been identified as YAP protein targets [49] (Fig. 3). Upstream components of the Hippo pathway that adversely inhibit YAP activity, such as Merlin, Expanded, Kibra, AMOTL2, and LATS kinases, are another family of transcriptional targets [50]. Thousands of new potential targets have been discovered according to genome-wide expression profiling and chromatin binding. However, there are significant discrepancies between the lists of targets discovered in research involving various cell types, implying that much of the YAP response is tissue or cell-type specific [50, 51]. The transcription of several genes involved in cell proliferation, differentiation, and growth could be affected by these relationships. In this part, we looked at how Hippo signaling interacts with other important pathways in leukemia.

### Hippo and Wnt signaling pathways

In HSCs, Wnt signaling is critical for maintaining homeostasis [52, 53]. Low levels of Wnt activation promote hematopoietic stem cell (HSC) function, whereas high Wnt doses reduce hematopoiesis, demonstrating that canonical Wnt signaling regulates hematopoiesis in a dose-dependent manner [54]. As a result, the HSC requires a precisely controlled quantity of Wnt signaling pathway activity for self-renewal, survival, growth, and proliferation [55]. The stimulation of Wnt signaling is a frequent, varied feature of all leukemia types. For example, individuals with FLT3-mutated AML have high amounts of β-catenin, promoting in vivo leukemia growth in xenograft mice reconstituted AML cell lines with del(5q) [56]. Furthermore, abnormal expression of Wnt pathway components such as WNT1, WNT2b, and LEF-1 is found in many AML cases, so Wnt signaling has a predictive value in AML [57].

The canonical Wnt pathway is CML’s most seriously impacted Wnt system [58]. Because the fusion protein BCR-ABL may actively adjust β-catenin levels in cells. In CML progenitors, nuclear β-catenin increased resistance to intrinsic tyrosine kinase inhibitor (TKI) [59]. In CML, FoxM1/ β-catenin interaction is essential for controlling canonical Wnt signaling and cancer stem cell self-renewal, proliferation, and tumorigenesis [60].

According to accumulating evidence, YAP/TAZ, the key effectors in the Hippo signaling cascade, regulate β-catenin levels and activity by physically interacting with β-catenin or Dvl. The first clear evidence that YAP/TAZ inhibited the Wnt/β-catenin pathway came from a study identifying TAZ’s direct interaction with Dvl in the cytoplasm. After Wnt3a stimulation, TAZ knockdown increased Dvl phosphorylation, consequently increasing the nuclear accumulation of β-catenin. Suppressing an upstream kinase in the Hippo pathway improved the connection between TAZ and Dvl, resulting in Wnt/β-catenin pathway downregulation [61]. TAZ’s role as a modulator of Wnt/β-catenin signaling is an interesting hypothesis. The β-catenin destruction complex, which is made up of APC, Axin, and GSK3, has been demonstrated to modulate TAZ levels. β-catenin phosphorylated by GSK3 functions as a scaffold for the interaction of TAZ with the TrCP E3 ligase complex in the absence of Wnt signaling [62]. Wnt3a was also discovered to cause TAZ dephosphorylation and stabilization, allowing TAZ to be more easily localized in the nucleus.

Because Wnt signaling is essential in leukemia stem cells and the microenvironment, targeting Wnt signaling pathways could help treat leukemia [63]. Wnt and Hippo signaling pathways control similar biological processes; therefore, they could regulate each other’s activity for precise systems biology rather than function [64]. Furthermore, YAP can be developed as a novel treatment target based on the two pathways by the intersection of these two signaling pathways (Fig. 3).

### Hippo pathway and mTOR interconnection

The cellular energy levels, amino acids, and other nutrients affect the rapamycin (mTOR) pathway [65]. Also, it is a master regulator of cell growth and metabolism and is an essential downstream effector of PI3K/AKT [66]. In recent trials, rapamycin and its analogs have shown significant anti-cancer activity in hematologic malignancies.

Given the importance of Hippo and mTOR signaling in growth control, it is not unexpected that links between them have been discovered. Mutation of the tuberous sclerosis complex (TSC), a critical negative regulator of mTORC1, resulted in an mTOR and autophagy-dependent overexpression of YAP proteins in a mouse cancer model [67]. mTORC2 phosphorylation reduces AMOT-YAP interaction, resulting in increased expression of YAP target genes [68]. In Drosophila, TOR suppression caused by genetic or dietary deficiency decreased Yki’s ability to access its target genes in the nucleus via an unknown mechanism [69]. Various signals can modulate the Hippo signaling in cancer stem cells, which are crucial in tumorigenesis.

### The Ras/MAPK and Hippo signaling pathways

This signaling pathway is critical for transmitting proliferative signals from receptors on membrane-bound [70]. In human cancers, RAS-MAPK pathway genes with canonical strong activating somatic mutations are observed in AMLs [71]. They could influence pathway components and upstream activators such as NRAS, KRAS, BRAF, PTPN11, and FMS-related tyrosine kinase 3 (FLT3), as well as chromosomal translocations in leukemia (for example, BCR-ABL and TEL-PDGFR) [72]. CRAF (RAF-1), BRAF, and ARAF are the three members of the RAF family of serine/threonine kinases [73], and RAF-1 has also been discovered to function in MAPK pathway activation and STK3, also known as MST-2, is a serine/threonine kinase that regulates apoptosis [73]. MST-2 is one of the most important components of the Hippo pathway in mammals [74]; besides, MST-2 and YAP/TAZ are essential Hippo pathway effectors that have been linked to melanoma cell metastatic and invasive abilities [73].

YAP has also been shown to affect how cancer cells respond to inhibitors of the MAPK pathway [75, 76]. Tumorigenesis is the result of a complex interaction between a number of variables and pathways [77] (Fig. 3). The RAF-1/MST-2 connection, according to studies, could be a novel link between the MAPK and Hippo pathways. Ras (or Ras-related molecules), Raf, MEK, and ERK inhibition may be useful in the treatment of leukemia [78]. Many inhibitors have been applied for clinical trials or are under consideration by the pharmaceutical industry to target essential components of this system [79] (Fig. 3).

### Hippo signaling pathway in leukemia

Deregulation of the Hippo signaling pathway is related to various solid tumors, including lung, breast, liver, and ovary [80]. Signaling pathways alteration can cause Leukemia, and among them, the Hippo pathway possesses significant effects on leukemia tumorigenesis [81]. The Hippo signaling pathway is an essential conservative pathway that helps regulate cell proliferation and apoptosis. Aberrant expression and mutation of core components in the Hippo signaling pathway such as MST1/2, LATS1/2, YAP, and TAZ easily promote cancer cell migration, invasion, and malignancy [82].

Many of the Hippo signaling pathway’s genes have been identified as tumor suppressors, such as MST1/2, SAV1, MOB1a/b, and LATS1/2, whereas others, such as YAP/TAZ, are oncogenes that stimulate malignant cells and allow them to proliferate uncontrollably [83]. Several studies have indicated that the activation of this pathway can be seen in many leukemia patients [84] (Table 1).

**Table 1:** Expression level of Hippo signaling pathway components in studies of leukemia

Hippo components	Expression level	Cancer type	No patients	Significant value	Samples	P value	References
Lats2	Overexpression	CML	67	Diagnosis marker, good prognosis and improve treatment response	PBMC	< 0.05	[104]
Aurka							
Taz							
Aurkb							
Mst1	No change	AML	52	–	PBMC	> 0.05	[105]
Mst2							
Yap1							
Mst1	Downregulation	Animal model of lymphoma and leukemia	–	Ability to prevent chromosomal instability	Lymphocytes	< 0.05	[106]
Yap	Overexpression	Leukemia and lymphoma	–	Proliferation	Jurkat cell line	< 0.05	[21]
Lats2	Overexpression	AML	32	Cancer development	PBMC	< 0.05	[86]
Yap	Overexpression	CML	–	Proliferation and leukemogenesis	BMMNCs	< 0.05	[26]
Lats2	Downregulation	ALL	101	Prognostic value	BMMNCs	< 0.05	[87]
Mobkl2a	Downregulation	MCL	77	Pathogenetic role for cancer development	Lymph node	< 0.05	[107]
Mobkl2b							
Lats2							

*AL* acute leukemia; *ALL* acute lymphocytic leukemia; *AML* acute myeloid leukemia; *AURKA/B* aurora kinase A/B; *BM-Mncs* bone marrow mononuclear cells; *CLL* chronic lymphocytic leukemia; *CML* chronic myelogenous leukemia; *LATS2*: large tumor suppressor kinase 2; *MST1* macrophage stimulating 1; *MCL* mantle cell lymphoma; *MOBKL2A* Mps one binder kinase activator-like 2A; *PBMCS* peripheral blood mononuclear cells; *SMZL* splenic marginal zone lymphoma; *YAP1* Yes-associated protein 1

YAP and TAZ are functional effectors that regulate gene expression by co-activating various transcription factors involved in leukemogenesis, such as RUNX, TEADS, and SMADS [81, 85]. In Gholami et al. the expression analysis of LATS2 as a tumor suppresser gene in de novo AML subjects has revealed that LATS2 may be correlated with leukemogenesis. *LATS2* gene was significantly overexpressed in patients who suffered AML compared to normal subjects [86] (Table 1). Another study revealed the *MST2-ETV6* fusion gene as a core component of the Hippo signaling system, a possible oncogene, in AML patients with t (8;12) translocation [81].

In line with AML, Acute lymphocytic leukemia (ALL) low expression of the *LATS2* gene was associated with ALL patients. Jimenez-Velasco et al. in their research, showed that low expression of the *LATS2* gene is linked to promoter region methylation in leukemia cells [87] (Table 1). MST1 deficiency has also been shown to enhance T-cell ALL in the presence of mutagenic stimulation in other studies. MST1 deletion mice also develop lymphomas faster, and lymphocytes have been found to have chromosomal instability. KIBBRA, a critical upstream component in the Hippo signaling pathway, is heavily methylated, and this is the crucial underlying leukemogenesis event in this subtype of leukemia [21].

Also, chronic lymphocytic Leukemia (CLL) studies demonstrated that YAP mRNA expression was more significant than healthy controls. In CLL, characterized as lymphoma with B cell accumulation in the blood, bone marrow, and lymph nodes, epigenetic modulation of WWC1 expression was also observed. The WWC1 gene was methylated in around one-third of CLL patients' samples, resulting in lower WWC1 expression [84]. YAP is overexpressed in patients' chronic myelogenous leukemia (CML) cells. Hui li et al. found that the expression level of YAP is significantly higher in CML patients’ bone marrow mononuclear cells, indicating that YAP plays a critical role in CML leukemogenesis. The result of another survey has revealed that LATS2 and AURKA, as well as TAZ and AURKB at advanced phases, are overexpressed compared to healthy control groups, which powerfully demonstrate the role of this signaling pathway deregulation in the pathogenesis of CML patients [26] (Table 1).

## Crosstalk between the Hippo pathway and miRNAs

MicroRNAs are highly involved in the Hippo pathway regulation. Several studies have shed light on the role of the Hippo pathway in tumorigenesis in various types of cancer such as breast, liver, gastric, glioblastoma cancers [88]. Importantly, miRNAs have been revealed to directly target and regulate the core components of the Hippo pathway. For example, miR-874-3p is significantly downregulated in colorectal cancer (CRC) tissue compared to normal tissues. MiR-874-3p by inhibition the YAP expression in the Hippo pathway resulting in the inactivation of the TEAD transcription [89]. Another research has revealed that miR-665 could promote proliferation and metastasis in hepatocellular carcinoma by inhibiting Hippo pathway activity [90]. In leukemia patients, which is the main topic of our article, several studies have been conducted to address the pivotal role of different microRNAs on the regulation of Hippo pathway components [91]. miR-550-1 acts as a tumor suppressor through the Hippo signaling pathway in AML. In a survey, microarray analysis revealed that miR-550-1 was significantly downregulated in the AML sample from the human patients, probably due to hypermethylation of the associated CpG islands. WWTR1 gene is considered a downstream target of miR-550-1, reducing the WWTR1 stability [92]. The information of the other microRNAs that contributed to the Hippo pathway in leukemia is described in Table 2.

**Table 2:** The contribution of microRNAs demonstrated to be involved in the Hippo pathway in leukemia

MicroRNAs	Expression level	Cancer type	Significant value	Samples	References
miR-9	Downregulated	AML	Activating Hippo/YAP signaling	Cell lines (THP-1, HL-60, TF-1, KG-1)	[108]
			Restrain the sharp increase boost apoptosis		
miR-550-1	Downregulated	AML	WWTR1 gene was a downstream target of miR-550-1	Cell lines (MV4-11, Kasumi-1 cells)	[92]
			Disrupted the proliferation and tumorigenesis of AML cells		
miR-181a	Downregulated	CML	Decreased activation of YAP		[109]
miR-7977	–	AML	miR-7977 inactivated the Hippo-YAP signaling pathway	Human BM CD34 + cells	[110]
			miR-7977 significantly reduced the expression of Hippo core kinase, STK4, YAP/TEAD		

*ALL* acute lymphocytic leukemia; *AML* acute myeloid leukemia; *CML* chronic myelogenous leukemia; *HL-60* human leukemia cell; *miR* microRNA; *STK4* serine/threonine kinase 4; *TEAD* transcriptional enhanced associate domain; *WWTR1* WW domain containing transcription regulator 1; *YAP* Yes-associated protein

## New pharmacological inhibitor targeting YAP

Despite advances in cancer treatment in recent decades, most patients respond poorly after a certain number of treatment cycles, and researchers also face significant challenges in treating cancer [93]. We summarize the pharmacological agents targeting the Hippo pathway to eliminate cancer cells. Dasatinib and statins represent compounds that inhibit YAP/TAZ activity via activating LATS [94]. Verteporfin represents compounds that inhibit the interaction between YAP/TAZ and TEAD [95]. Blebbistatin, Botulinum toxin C3 and LY294002PDK1 inhibitor II inhibit YAP/TAZ nuclear localization and transcriptional activity [96, 97]. Discoveries imply the suppression of YAP/TAZ-driven transcription via CDK9 inhibitors [98].

**Fig. 4:**
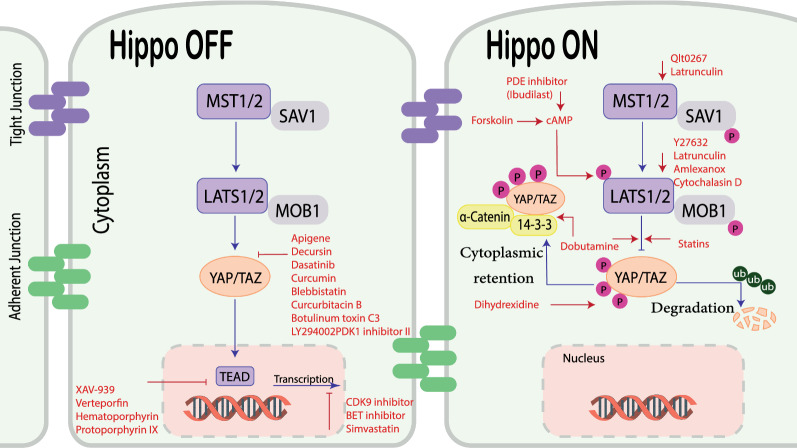
New druggable agent effective in targeting YAP. The figure shows the drug agents that can be effective in the Hippo signaling pathway. Some of these drugs inhibit YAP/TAZ nuclear localization or block the transcription of target genes. Some others lead to the degradation of these proteins by increasing LATS activity and phosphorylation of the YAP/TAZ complex. *LATS* large tumor suppressor kinase; *Yap* Yes-associated protein

Simvastatin also has a potent YAP/TAZ inhibiting action. Ibudilast (a PDE4 selective inhibitor) and Forskolin can promote YAP phosphorylation by preventing cAMP breakdown, implying that PDE inhibitors may be useful in the treatment of cancers with YAP oncogenic activity [99, 100]. Dobutamine’s possible anti-cancer activity was recently investigated in a variety of cancer types. Dobutamine causes phosphorylation of YAP-Ser127, which causes YAP-dependent gene transcription to be suppressed [101]. Latrunculin B and cytochalasin D, which disrupt the actin cytoskeleton, limit YAP activation in response to cell attachment to the ECM-Inhibition of nuclear YAP localization via increased LATS activity [102]. Dihydrexidine increases YAP phosphorylation and inhibits Hippo signaling pathway [103] (Fig. 4).

## Conclusion and future perspective

Hippo signaling plays an important role in tumor initiation, invasion, drug resistance, metastatic potential, and self-renewal of cancer stem cells, as well as developmental control. According to studies on this signaling pathway, YAP as a tumor suppressor gene can be involved in many types of cancer. In leukemias, although not much information is available, the increased expression of this protein shows a significant relationship with the poor prognosis of patients. Therefore, the study of the mechanism of action YAP and the factors affecting its inhibition in cancer can be proposed as new pharmacological agents in leukemia treatment.

## Data Availability

The primary data for this study is available from the authors on direct request.

## Abbreviations

Akt: Protein kinase B
ALL: Acute lymphocytic leukemia
AML: Acute myeloid leukemia
AMPK: AMP-activated protein kinase
APC: Adenomatous polyposis coli
APL: Acute promyelocytic leukemia
AURKA/B: Aurora kinase A/B
CRC: Colorectal cancer
CLL: Chronic lymphocytic leukemia
CML: Chronic myelogenous leukemia
DVL: Dishevelled
ERK: Extracellular signal-regulated kinase
FOXH1: Forkhead box H1
GLUT3: Glucose transporter 3
GSK3B: Glycogen synthase kinase 3 beta
HCC: Hepatocellular carcinoma
HSC: Hematopoietic stem cell
JNK: Jun N-terminal kinase
LATS2: Large tumor suppressor kinase 2
LEF1: Lymphoid enhancer binding factor 1
LKB1: Liver kinase B1
MAP: Mitogen-activated protein
Mats: Mob as tumor suppressor
MEK: MAPK/ERK kinase
MOB1a/b: MOB kinase activator 1A/B
MOBKL2A: Mps one binder kinase activator-Like 2A
MST1/2: Macrophage stimulating 1/2
mTOR: Mammalian target of rapamycin
KIBRA: Kidney and brain protein
PI3K: Phosphatidylinositol-3-kinase
PTEN: Phosphatase and tensin homolog
Sav: Scaffold protein salvador
SMAD: Mothers against decapentaplegic homolog
STK4/3: Serine/threonine kinase 3/4
TAZ: Tafazzin
TAZ: Transcriptional co-activator with PDZ binding motif
TCF: T-cell factor
TEAD1-4: TEA DNA-binding proteins
TGF: Transforming growth factor
Wts: Protein kinase warts
WWTR1: WW domain-containing transcription regulator protein 1
YAP: Yes-associated Protein
Yki: Yorkie
